# Consumer-oriented (wearable) sleep technology: a systematic SWOT analysis and recommendations for athletes and fitness enthusiasts

**DOI:** 10.3389/fspor.2026.1872518

**Published:** 2026-07-10

**Authors:** Kristina Klier, Lukas Masur, Peter Düking

**Affiliations:** 1 Faculty of Computer Science, Institute of Computer Engineering, University of the Bundeswehr Munich, Neubiberg, Germany; 2 Department of Sports Science and Movement Pedagogy, Technische Universität Braunschweig, Braunschweig, Germany

**Keywords:** consumer technology, sleep, sleep assessment, sports, tracking, wearables

## Abstract

Sleep is a key determinant of health, recovery, and performance, yet sleep disturbances are prevalent in both athletic and general populations. With the rapid expansion of consumer-oriented (wearable) sleep tracking technologies, this study aimed to systematically synthesize current evidence and derive best-practice recommendations for their use in physically active populations. A systematic literature search (PubMed, Scopus, EBSCO) identified 21 eligible studies, which were analyzed using a strengths, weaknesses, opportunities, and threats (SWOT) framework. In total, 9 strengths, 12 weaknesses, 8 opportunities, and 8 threats were extracted. When applied correctly and longitudinally, consumer wearables can provide meaningful insights and support sleep self-management, however, if applied incorrectly, consumer wearables risk misinterpretation and inappropriate training or recovery decisions. Future research should prioritize rigorous validation, and context-specific applications, alongside development and evaluation of user education strategies to ensure optimal use of consumer-oriented (wearable) sleep technologies.

## 1 Introduction

Optimal sleep is fundamental for athletic recovery, health, and peak performance (1–3). Insufficient or disturbed sleep can impair mood, cognitive function, and physical performance, while increasing the risk of injury and illness (4–6). For example, in their systematic review, Craven and colleagues (7) have calculated an average performance decline of −0.4% per hour awake when nocturnal sleep is deprived (≤6 h of sleep within a 24-hour period). Research shows that many athletes struggle to obtain adequate sleep. Elite athletes, for instance, often sleep less than the recommended 7–9 h (8) and experience poor sleep quality (9–11). Consequently, sports science experts emphasize prioritizing and optimizing sleep as a key component of training and recovery at all levels (12–15). Technical advancements are of constantly increasing usage and intend to overcome (previous reported) sleep related issues. While polysomnography (PSG) represents the gold standard in tracking sleep behavior and patterns, this complex laboratory measurement is often too cumbersome for the integration in everyday life (16). Mobile sleep tracking has emerged as a prominent tool for monitoring sleep patterns and understanding individual sleep behavior in free-living conditions (17, 18). Consumer technologies, such as wearable devices and smartphone applications, claim to allow for continuous, non-invasive measurement of various sleep parameters (e.g., sleep duration, sleep efficiency, and sleep stages) (19, 20). However, the accuracy of these devices can vary depending on the technology employed, and the interpretation of data remains challenging, particularly in distinguishing between normal variations in sleep and clinically significant disorders (21–23). These devices are in widespread public use and continue to evolve rapidly, offering opportunities for personalized sleep feedback and early detection of potential problems when used judiciously (24). This juxtaposition of high consumer interest and uncertain scientific validity highlights the need for clear guidance on how to effectively utilize consumer-oriented (wearable) sleep technologies in practice. Notably, in professional sports settings dedicated staff may help collect and interpret sleep data, whereas recreational athletes often lack such support and must interpret their own data. This situation underscores a critical gap: the absence of structured, evidence-based recommendations for leveraging consumer-oriented sleep technology outside of elite programs or formal research validations. In light of these challenges, there is a compelling need for an evidence-driven resource to guide non-elite athletes and fitness enthusiasts in using sleep tracking technologies effectively. To provide such a resource, and in line with research on other emerging technologies (25–27), this article aimed to provide a comprehensive strengths, weaknesses, opportunities and threats analysis (SWOT analysis) of consumer-oriented sleep technology which can be used to derive best-practice recommendations for the handling of these technologies for amateur athletes or fitness enthusiasts.

## 2 Materials and methods

### 2.1 Study design

We outline the strengths (S), weaknesses (W), opportunities (O), and threats (T) of consumer-oriented (wearable) sleep tracking technology. Besides a summary of evidence, we aimed to derive best-practice recommendations for the handling of consumer-oriented sleep technology in sports. This SWOT analysis was based on a systematic literature search. The search protocol was based on Preferred Reporting Items for Systematic Reviews and Meta-Analyses (PRISMA) guidelines (28).

### 2.2 Search strategy and eligibility criteria

The systematic literature search was conducted on July 14th 2025 using three databases: PubMed, Scopus, and EBSCO (no filters applied). Search terms were defined with a skilled librarian (see Table 1).

**Table 1:** Search strings (terms within each variable combined using AND).

Variable	Search strings
Population	(“athlet*”)
Device	(“consum* technol*” OR “wear*” OR “track*” OR “monitor*” OR “device*”)
Method	(“assess*” OR “track*” OR “monitor*” OR “evalu*”)
Outcome	(“sleep*”)

Eligible articles needed to be peer-reviewed and written in English. Studies without available full text were excluded. Included populations were healthy, physically active populations, respectively athletes of all sports and any gender aged 18–60. Non-human, clinical, or ill populations were excluded. Sleep should be assessed via smartphone, apps or wearables. Wearables were defined as devices that are worn on the body recording bio-signals (e.g., heart rate, body temperature) and further information (e.g., acceleration data) (29). Other technologies such as smart mattresses etc. were excluded. Regarding the study type, monitoring and validation studies as well as any kind of reviews and meta-analysis were included. Studies focusing solely on sleep characteristics or sleep (hygiene) interventions were excluded.

Studies identified through the electronic search were exported and imported into Catchii (30), which was subsequently used for duplicate removal and the review process. The eligibility of titles and abstracts were examined by one reviewer (KK). Full texts were then screened independently by two reviewers (KK, LM). A third investigator (PD) resolved any discrepancies if necessary.

### 2.3 Risk of bias, data extraction and synthesis

Risk of bias assessment was not applicable for the non-systematic reviews and the survey study. Risk of bias assessment of the systematic review was performed adapting the Cochrane ROB-ME tool (31). The Cochrane ROB2 tool (32) was used for risk of bias assessment of the randomized controlled trial (RCT) and the study which was part of a RCT. The Cochrane ROBINS-I tool (33) was the basis for the risk of bias assessment of the remaining included studies. These assessments as well as data extraction were examined by the main investigator (KK). The following items were extracted: first author's name, publication year, population, context/measures, and main results (see Supplementary Table S1). The extracted data was thematically analyzed and structured into SWOTs. This hermeneutic coding strategy consisted of three stages: 1) initial coding—remaining open to all possible themes indicated by initial readings of the included studies (34, 35), 2) focused coding—categorizing the data inductively based on thematic similarity (34), and 3) theoretical coding integrating thematic categories (36). After ordering key themes according to their frequency in the literature, they were assigned to the categories strength, weakness, opportunity, threat based on discussion within the research team. The first coding stage was performed by one reviewer (KK), stage 2 by two reviewers (KK, LM), and the third investigator (PD) resolved any discrepancies if necessary. The third coding stage was performed several times: Every investigator independently assigned the categories; these were then overlaid and discrepancies were resolved through several rounds of discussion. For category assignment, we differentiated between technical (e.g., hard-/software) and content-related (e.g., presentation of respectively working with sleep data) aspects as well as negative (W/T) and positive (S/O) connotation in the included studies.

## 3 Results

### 3.1 Literature search

**Figure 1:**
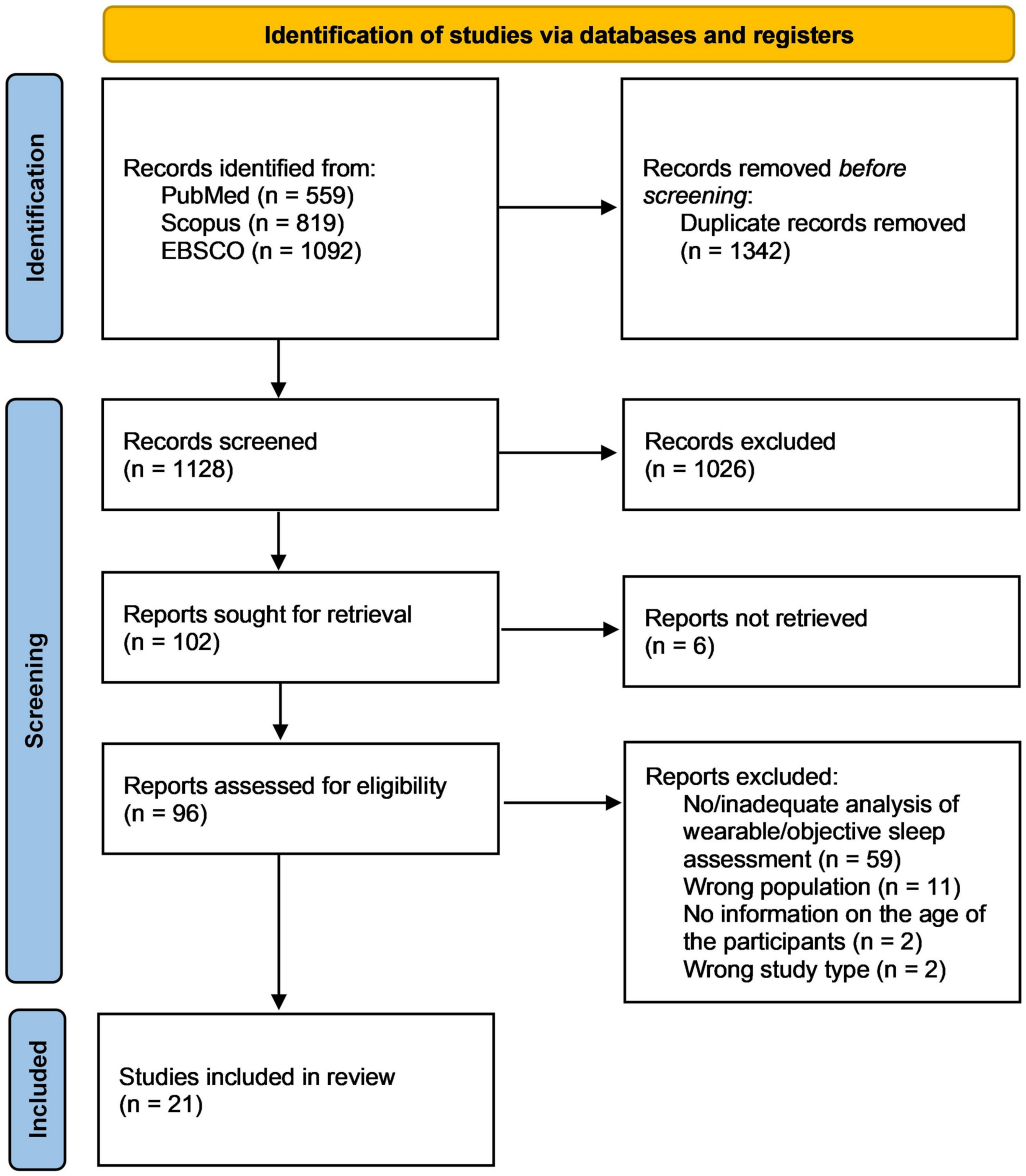
PRISMA flow chart.

Figure 1 presents the PRISMA flow chart of the search process. A total of 2,470 records were retrieved and 1,342 duplicates removed. Finally, 21 studies were included in this systematic review and SWOT analysis.

The included studies are summarized in Supplementary Table S1. Of these, one study was a systematic review with meta-analysis (37), eleven were non-systematic/narrative reviews (38–48), and nine were intervention studies (one RCT (49), one study being part of a RCT (50), one survey (51), six observational studies (52–57)). The systematic review with meta-analysis as well as the RCT and the validation studies conducted in a sleep laboratory were judged to have a low risk of bias. Whereas all other included studies were judged to have some or even serious concerns for overall risk of bias. Nine strengths, twelve weaknesses, eight opportunities, and eight threats were extracted from the included studies (see Table 2).

**Table 2:** Extracted SWOTs with references.

STRENGTHS	WEAKNESSES
– Increasing availability to consumers as number of available devices (including a variety of sensors) is growing (46, 41, 40, 48, 43, 47, 42, 44, 55, 57) – Higher accuracy in sensing sleep/wake when using different signals (most common: HR, HRV, respiratory pattern, motion) (38, 46, 40, 43, 47, 42, 44, 55, 57) – Practical, effective, non-invasive, cost-effective, time-effective (38, 39, 50, 41, 40, 48, 47, 42, 44) – Ability to provide direct/individualized feedback (38, 41, 48, 51, 49, 42, 44, 55) – Sleep monitoring in individuals’ own environments (38, 50, 37, 56, 53, 48, 45, 44) – Potential to assess qualitative and quantitative sleep parameters (38, 39, 37, 40, 48, 47, 45, 55) – Objective measurement method (most popular wearing location: wrist) (50, 37, 40, 43, 47, 42, 55) – Many wearables integrate health ecosystems by syncing with dedicated mobile app or Web-platform (40, 48, 43, 42, 44) – Portable PSG/neurophysiological-based devices as economical and time-friendly to assess EEG related parameters compared to more sophisticated devices (53, 57)	– Lack of validation studies for most devices (38, 46, 37, 41, 40, 48, 43, 42, 44, 57) – Different manufacturer specifications and unknown algorithms limit reliability and sensitivity of measurements (38, 37, 41, 40, 48, 43, 42, 44) – Overestimation of sleep and underestimation of wake as actigraphy devices rely on motion (50, 37, 41, 43, 47, 54, 42, 44) – Lowest evidence for tracking sleep stages (due to methodological difficulties with sleep staging) (37, 40, 48, 43, 54, 42, 44) – Expertise needed to interact and interpret data correctly (46, 41, 47, 42, 45, 55) – Different calculations/definition of sleep parameters depending on device/manufacturer limit standardization and comparability of measured parameters (39, 46, 37, 44, 55) – Lack of medical devices (46, 40, 54, 42) – Assessment of sleep parameters by approximation from indirect parameters (43, 42, 44) – Critical privacy and security concerns (e.g., third-party data sharing, data collection purposes, user control and rights) (41, 42, 44) – Compatibility with Android/iOS may be limited (38, 44) – No international consensus/lack of best practices for validation studies (43, 44) – PPG data can be compromised due to skin tone or tattoos (43)
OPPORTUNITIES	THREATS
– To monitor regularly and longitudinally in different settings and locations (50, 37, 41, 56, 40, 53, 48, 54, 42, 45, 52, 44, 57) – To gain more holistic insights into performance and health by sleep monitoring (38, 37, 41, 56, 40, 48, 47, 51, 54, 45, 52, 55) – To detect health problems or overtraining early (39, 56, 47, 54, 45, 52, 44, 57) – To collect objective data which can/should be combined with subjective data (38, 39, 37, 40, 53, 51, 52, 55) – To raise awareness and promote healthy sleep habits (46, 41, 40, 48, 51, 49) – To influence individuals’ knowledge and attitude about their own sleep health (46, 41, 51, 49) – To combine tracking and feedback systems in one technology to reduce technological burden on individuals (41, 40, 49, 44) – To support self-management/self-control (51)	– To conclude misleading recommendations for recovery and training due to poor sleep data quality (38, 39, 46, 41, 48, 49, 42) – Threat of Orthosomnia due to excessive monitoring (46, 41, 51, 42, 52) – Misconceptions about “healthy sleep” enforced by wearable data (e.g., falling asleep immediately) (46, 41, 48, 51) – Frequent/daily feedback might lead to overinterpretation of single data points (41, 51, 49, 42) – Commitment/adherence necessary for longer monitoring periods (54, 45, 52, 57) – Potential that device impairs perception of sleep based on erroneously measured sleep parameters (46, 41, 51, 42) – Interpretation of sleep parameters without additional contextual information might be insufficient to alter decisions/course of action/sleep behavior (41, 48) – Growing number of devices and sensors might result in overload and impairs selection of a specific device potentially leading to selection of wrong/false device for a specific scenario/purpose (46)

In total, 37 extracted aspects; 4 times double assignment due to positive + negative interpretation.; HR, Heart Rate; HRV, Heart Rate Variability; PPG, Photoplethysmography; PSG, Polysomnography.

### 3.2 Strengths

Of the overall extracted 37 aspects, nine were clustered as strengths. These were extracted of 19 out of the 21 studies included. The most often mentioned strength (*n* = 10) is the increasing availability of consumer-oriented wearable technology (40–44, 46–48, 55, 57). In particular, the number of available devices as well as the number of built-in sensors are constantly increasing. Likewise, *n* = 9 studies reported that the sleep measurement accuracy improves when additional sleep-relevant parameters are simultaneously integrated into the device algorithm (38, 40, 42–44, 46, 47, 55, 57). Another often named (*n* = 9) strength is the practicability and efficiency of apps and wearables: They are easy to apply, non-invasive, cost- and time-effective (38–42, 44, 47, 48, 50). Eight studies mentioned the ability to provide direct and individualized feedback (38, 41, 42, 44, 48, 49, 51, 55), the (daily) sleep monitoring in the individuals' environments (i.e., outside the laboratory) (37, 38, 44, 45, 48, 50, 53, 56), and the potential to assess qualitative and quantitative sleep parameters (37–40, 45, 47, 48, 55) as further strengths. According to *n* = 7 studies, wrist-worn technology is most common as objective measurement method (37, 40, 42, 43, 47, 50, 55). Five studies described the strength of wearables in integrating health eco-systems by syncing with the dedicated mobile app or Web-platform (40, 42–44, 48). *N* = 2 studies reported that portable polysomnography (PSG) systems, as an alternative to activity-based devices, are gaining relevance. These systems appear to be similarly cost-efficient and user- and time-friendly. However, their main advantage lies in the inclusion of electroencephalography (EEG), which enables assessment of sleep architecture, including reliable sleep staging.

### 3.3 Weaknesses

In total, 12 weaknesses were extracted out of 16 studies. The most often mentioned aspect (*n* = 10) is the lack of validation studies for most devices (37, 38, 40–44, 46, 48, 57). Eight studies reported manufacturer related weaknesses (37, 38, 40–44, 48): Whilst it is known that the applications' and devices' specifications can differ between manufacturers or even between versions, the data processing of the measurements often remains a black box. Often, verifying if the algorithm has been modified by the manufacturer remains difficult without direct access to the algorithm. Thus, the unknown algorithms limit the reliability and sensitivity of the measurements. Moreover, *n* = 8 studies specified that, when devices rely on motion, they tend to overestimate sleep and underestimate wake (37, 41–44, 47, 50, 54). Seven studies mentioned that the evidence is lowest for tracking sleep stages due to methodological difficulties with sleep staging (37, 40, 42–44, 48, 54). According to *n* = 6 studies, an additional weakness to sustainably improve individuals' sleep is the required expertise for interaction and interpretation of sleep data (41, 42, 45–47, 55). Five studies stated that the overall available number of measurements as well as the calculations respectively definitions of sleep parameters vary between devices or manufacturers and thus, limit the standardization and comparability of measured parameters (37, 39, 44, 46, 55). *N* = 4 studies named the lack of medical devices for sleep tracking in free-living settings as further weakness (40, 42, 46, 54). Three studies each highlighted that actigraphy, wearables, and apps do only measure sleep indirectly (i.e., no direct measurement but approximation of sleep parameters) (42–44) and that privacy and security concerns seem critical aspects as sleep data are highly sensitive which is why a secure collection and storage should be guaranteed (41, 42, 44). Two studies each described that the usability is further limited when compatibility is not given with both iOS and Android (38, 44) and that there is no standard approach on applying and researching consumer-oriented sleep tracking technologies (43, 44). With regard to the missing validation studies, the literature is lacking in best practices and international consensus statements (44). Lastly, *n* = 1 study stated to take into account that the PPG sensor technology (photoplethysmography; which is installed in most wearables) has problems across skin tones: The heart rate detection seems less accurate in no-white skin tones or individual with tattoos which in turn can lead to measurement errors (43).

### 3.4 Opportunities

*N* = 8 opportunities were extracted of 20 out of the 21 studies included. The most often mentioned aspect (*n* = 13) is the opportunity to monitor regularly and longitudinally in different settings and locations (i.e., at home, during training camps or competitions) (37, 40–42, 44, 45, 48, 50, 52–54, 56, 57). *N* = 12 studies emphasized that sleep tracking can be part of a holistic performance and health monitoring strategy (37, 38, 40, 41, 45, 47, 48, 51, 52, 54–56). In contrast, *n* = 8 studies reported that this approach enables early detection of health problems or symptoms of overtraining (39, 44, 45, 47, 52, 54, 56, 57) and facilitates the collection of objective data that can be combined with subjective measures to provide more comprehensive insights (e.g., diary plus sensor/wearable) (37–40, 51–53, 55). According to *n* = 6 studies, sleep tracking technology enables increased awareness and even promotes healthy sleep habits (40, 41, 46, 48, 49, 51). Four studies each stated that the devices and apps have the potential to influence the individuals' sensitivity and attitude about their own sleep health by broadening their knowledge and providing various insights in their sleep patterns (41, 46, 49, 51). An additional opportunity is the combination of tracking and intervention in one technology, i.e., it is possible to get not only feedback but also use tailored interventional features such as “smart alarm” (40, 41, 44, 49). At last, *n* = 1 study resumed that the technology with all its features and specifications provides the opportunity to support the individuals' self-management respectively self-control (51).

### 3.5 Threats

Overall, eight aspects were clustered as threats. These were extracted out of 11 studies. The most often mentioned aspect (*n* = 7) is the threat to conclude misleading recommendations for recovery and training due to poor sleep data quality (38, 39, 41, 42, 46, 48, 49). Five studies reported that excessive monitoring can lead to orthosomnia (i.e., pursuit of optimal sleep that is driven by sleep tracker data) (41, 42, 46, 51, 52). Four studies each described that some could conclude misconceptions about “healthy sleep” enforced by wearable data (e.g., falling asleep immediately) (41, 46, 48, 51) as well as that the frequent/daily feedback provided by apps and wearables might lead to overinterpretation of single data points (41, 42, 49, 51). *N* = 4 studies reported that, particularly during longer monitoring periods, high user commitment and adherence are required (45, 52, 54, 57). At the same time, to avoid so-called “techno-stress” associated with continuous tracking, it is important to ensure that technology does not replace athletes' own bodily sensations and self-awareness (41, 42, 46, 51). *N* = 2 studies claimed that it seems important to take the typical 24 h into account when interpreting the sleep data as the interpretation of sleep parameters without additional contextual information might be insufficient to alter decisions/course of action/sleep behavior (41, 48). Finally, as mentioned in *n* = 1 study, the growing number of devices and sensors might result in overload and impairs selection of a specific device potentially leading to selection of wrong/false device for a specific scenario/purpose (46).

## 4 Discussion

The main aim of this study was to provide a comprehensive strengths, weaknesses, opportunities and threats analysis (SWOT analysis) of consumer-oriented (wearable) sleep tracking technology. We identified *n* = 9 strengths (most often mentioned included amongst others: growing number of sensors/available devices, high feasibility, direct feedback), *n* = 12 weaknesses (most often mentioned included amongst others: lack of validation as well as medical devices, unknown algorithms), *n* = 8 opportunities (most often mentioned included amongst others: holistic/longitudinal health and performance monitoring, early detection of health problems or overtraining, combination of objective and subjective data), and *n* = 8 threats (most often mentioned included amongst others: risk of orthosomnia, misconceptions about “healthy sleep”, overinterpretation of single data points).

### 4.1 Strengths and opportunities

Traditional laboratory-based assessments require complex setups, specialized equipment, and trained personnel, making them resource-intensive. In addition, the so-called first-night effect, where sleep measurements obtained in an unfamiliar environment are not representative, further limits the feasibility and validity of sleep laboratory assessments (58). These limitations are increasingly mitigated by consumer-oriented wearable technologies. Such devices enable longitudinal sleep monitoring in familiar environments and during real-life contexts such as training and competition, without interfering with the individual's routine. From a practical perspective, wearable devices are characterized by high accessibility. Their small sensors are comparably easy to apply and typically require only a smartphone for data synchronization. This allows flexible, on-demand, and repeated assessments of individual sleep patterns. Beyond usability, wearable technology offers advantages for more holistic monitoring. Many devices integrate the assessment of activity and recovery within a continuous 24-hour cycle using a single system. This is particularly relevant for athletes and physically active individuals, as it enables comprehensive monitoring of both health and training status (59, 60). Furthermore, recent technological developments extend beyond passive monitoring by providing individualized recommendations for recovery and training (61). However, the recommendations require careful inspection, interpretation and contextualization before application in practice. From a behavioral perspective, sleep tracking represents a prerequisite for subsequent interventions. Before implementing sleep hygiene or optimization strategies, individuals can develop awareness of their own sleep and recovery patterns by relying on wearable technologies providing continuous, personalized feedback on sleep behavior. However, methodological considerations remain relevant. In line with established actigraphy recommendations, at least 72 h of continuous monitoring are required to obtain reliable outcome measures (62). In addition, wearable-based measurements appear to follow a learning curve, with accuracy improving as the duration of device usage increases (63). Finally, ongoing advancements in device technology and the increasing number of integrated sensors are likely to further enhance measurement accuracy over time.

### 4.2 Weaknesses and threats

The validity of consumer-oriented wearable sleep tracking technologies remains insufficiently investigated, which limits the reliability of the data they provide (64, 65). This is also due to the fact that the validation studies differ not only in their methodology but also in the metrics used [see (66–68) for detailed treatment of key validation concepts such as sensitivity, specificity, agreement with PSG, or epoch-by-epoch accuracy]. This highlights the need for more high-quality validation studies to establish robust evidence on measurement validity. To enable meaningful interpretation of wearable-derived sleep data, it is necessary to contextualize these data with additional information, such as physical activity, nutrition, and environmental factors (69). Without such contextualization, there is a risk of misinterpreting isolated data points, particularly in cases of measurement errors or missing data, which may lead to erroneous conclusions. Measurement limitations further complicate data interpretation. Sleep fragmentation has been shown to increase missing data rates and reduce measurement accuracy (70). In addition, commonly used accelerometer- and photoplethysmography (PPG)-based technologies exhibit limitations in accurately detecting sleep stages (71). Consequently, wearable devices typically demonstrate high sensitivity (i.e., detecting wake) but lower specificity (i.e., detecting sleep), increasing the likelihood of over- or underestimation of sleep parameters (72). In practical terms, such inaccuracies may negatively affect recovery-related decisions. These limitations can be explained by underlying sleep physiology. Sleep stage classification primarily depends on characteristic brain wave patterns, which define transitions between light, deep, and REM sleep (73). Physiological signals such as heart rate or movement are secondary responses to these neural processes. Therefore, technologies directly assessing brain activity, such as EEG-based systems (e.g., smart headbands), provide higher accuracy compared to motion- or PPG-based approaches (74). Additional technological constraints must also be considered. PPG-based sensors show reduced accuracy across different skin tones and in individuals with tattoos, which may compromise data quality and generalizability (75, 76). Regarding future research, there is not only a need for validation studies but also for examining different technologies in different target-groups and settings. Furthermore, a standardized validation approach as well as customizable digital sleep interventions for athletic populations should be developed. Ethical considerations, particularly regarding data privacy and security, represent an important limitation when applying consumer-oriented wearable sleep tracking technologies in practice (77, 78) and should be assessed more often.

While use of consumer-oriented wearable technology can raise awareness of sleep related parameters, it can however hold the risk of adopting false beliefs about healthy sleep (79). For instance, falling asleep quickly and sleeping through the night might not generally be healthy, it can also be a sign of overtraining or (work) overload and insufficient recovery. It should be mentioned that the goal is not to “optimize sleep related parameters”, but sleep (80). Notably, recent research provides evidence that individuals can experience pressure to track and optimize which in turn can lead to techno-stress (81, 82) or orthosomnia (83, 84). Finally, prior to the application of consumer-oriented wearable sleep technology, it is essential to consider the reason for and possible scope of tracking (85, 86). To avoid a flood of data and to be able to process respectively handle the data sustainably, a decision-making framework can be employed using the questions (68, 87): *1)* Would the promised information be helpful? *2)* Can you trust the information you will be getting? *3)* Can you integrate, manage and analyze the data effectively? *4)* Can you implement the technology in your practice? Acknowledging that other aspects might be relevant, only if (at least) these questions can be answered with a yes, implementations of consumer-oriented sleep technology should be considered.

### 4.3 Aspects which could be an opportunity and a threat

Depending on the connotation respectively described context, four aspects could not only be assigned in one SWOT category. Those double assignments referred to devices/sensors, feedback, environment, and knowledge. On the one hand, the variety of devices and built-in sensors constantly broaden the number of parameters measured with increasing accuracy. This offers multiple settings and application for private use as well as for research (88, 89). On the other hand, this might lead to overload and makes it more difficult to choose the appropriate device. While scientists currently try to establish databases and frameworks to provide user-oriented information (90–92), individuals might be overwhelmed in their search and decision for the right device. When using a wearable or a smartphone app, a major advantage is the ability to receive real-time and personalized feedback on the measured parameters, and thus, directly adjust one's behavior (93, 94). In persons who are either easily unsettled or trust the data more than their feelings, the frequent/daily feedback might lead to uncertainty or overinterpretation of single data points (95, 96). Regarding the setting for consumer-oriented (wearable) sleep tracking, the advantage is twofold: individuals can either sleep and track at home, i.e., in their familiar environment, or on-demand mobile, i.e., in training camps or at competitions. The threat here is to deal with the limited accuracy of non-laboratory measurements. Thus, if a clinically relevant assessment of sleep or health issues is required, at least a medical device should be used, or a sleep laboratory should be consulted (85, 97). Lastly, consumer-oriented (wearable) sleep technology has the potential to influence individuals' knowledge and attitudes about their own sleep and thereby support a healthy lifestyle and improve sleep health literacy. But if data and its implications are misunderstood, a negative consequence might be that individuals develop a misguided understanding of what “good sleep” is and establish dysfunctional sleep-related routines (79, 98).

### 4.4 Recommendations to mitigate weaknesses and threats, and leverage strengths and opportunities

Sleep represents a fundamental biological process underpinning recovery, health, and human performance. Accordingly, sufficient and restorative sleep should be actively prioritized and supported, which necessitates a basic understanding of sleep physiology and its determinants. If individuals intend to utilize consumer-oriented technologies to monitor or optimize sleep, a critical evaluation of device quality is essential. While different aspects can be taken into consideration when defining “quality” of a wearable, this includes e.g., reliability and validity, but also aspects such as usability, comfort, device placement, sensor configuration, and cost (68). Users should further evaluate frequency of provided feedback, and if devices “only” monitor or aim to provide recommendations on improving aspects of sleep, ensuring alignment with individual needs, and develop a sound understanding of the reported sleep parameters and their underlying physiological mechanisms (99).

While different parameters can be obtained from consumer-oriented (wearable) technologies, users should select the devices and parameters which have shown to be valid and reliable (such as e.g., sleep duration, sleep regularity), and which can be interpreted and ideally inform decision-making to improve sleep. Pseudo-parameters should be interpreted very cautiously (100). It seems recommendable that sleep data should not be overinterpreted based on single observations. Instead, longitudinal monitoring is recommended, for example by analyzing rolling averages of sleep duration over periods of 14, 21, or 28 nights, to account for natural variability (42, 62). Key sleep parameters considered suitable for longitudinal monitoring include total sleep time (TST), sleep efficiency (SE), and regular sleep timing. When available, sleep onset latency (SOL) and wake after sleep onset (WASO) may also be included (37, 101, 102). Objective data should be interpreted in conjunction with subjective sleep perception, as reliance on device-derived metrics alone may be misleading (39, 52, 103). Where possible, sleep-related information should be contextualized with additional variables such as nutritional status, training load, travel, and psychosocial stressors to enable more meaningful interpretation and actionable insights (41, 48, 69). Furthermore, maintaining up-to-date firmware and software is necessary to ensure optimal device performance, acknowledging that this might affect e.g., reliability and validity of provided data (104, 105). In cases of uncertainty, consultation with qualified experts is advisable to support appropriate interpretation and application of sleep-related data. A summary of these best-practice recommendations is provided in Table 3.

**Table 3:** Best-practice recommendations at a glance.

Best-practice recommendation	Practical implication
Prioritize sleep as a biological recovery process	Sleep should be treated as a fundamental determinant of recovery, health, and performance. Users need (basic) knowledge of sleep physiology and relevant determinants.
Critically evaluate device quality before use	Select devices based on e.g., reliability, validity, usability, comfort, placement, sensor configuration, cost, and compatibility with the intended use case.
Match device functions to individual needs	Users should check whether the technology only monitors sleep or also provides recommendations, and whether the feedback frequency is appropriate for the user.
Select valid and interpretable parameters	Focus on parameters with acceptable evidence for validity and reliability. Sleep duration and sleep timing are generally more interpretable than derived or proprietary scores. Pseudo-parameters should be interpreted cautiously.
Avoid overinterpretation of single nights	Single-night deviations should be interpreted cautiously. Sleep data should be interpreted longitudinally.
Use rolling averages	Analyze trends over 14, 21, or 28 nights to account for natural night-to-night variability.
Combine objective and subjective data	Device-derived sleep data should be interpreted together with subjective parameters such as e.g. sleep perception, perceived recovery, fatigue, mood, and well-being.
Contextualize sleep data	Interpretation should include relevant contextual factors such as training load, nutrition, travel, competition, psychosocial stress, illness, and environmental conditions.
Maintain device software and firmware	Regular updates are necessary because algorithmic changes may affect data quality, reliability, and validity.
Seek expert support when needed	In cases of uncertainty, qualified experts should support interpretation and application of sleep data, especially when data influence training, recovery, or health-related decisions.
Avoid over-reliance on wearable data	Wearable data should support, not replace, body awareness, subjective perception, and professional judgment.
Use sleep data to inform, not dictate, interventions	Sleep-related interventions should be based on longitudinal patterns, subjective-objective agreement, and individual context rather than isolated wearable outputs.

### 4.5 Study strengths and limitations

This SWOT analysis demonstrates methodological strength, as its findings are based on a systematic literature search across three databases. In addition, it offers practical recommendations that are accessible and readily applicable for users of wearable devices and mobile applications. However, several limitations must be acknowledged. The *a priori* defined search terms and the focus on sport-specific contexts may have led to the exclusion of relevant studies addressing sleep assessment in non-sport or broader real-world settings. Furthermore, the transferability of findings derived from elite populations to recreational or general populations should be performed cautiously. The risk of bias assessment indicated some concerns across the included studies, which may reduce the external validity of the synthesized evidence. In addition, this analysis did not differentiate between wearable placement sites, although, depending on the specific parameter at question, device location is known to influence measurement accuracy (106). The same applies to the analyzed consumer-oriented sleep technology. This SWOT analysis provides a comprehensive overview of strengths, weaknesses, opportunities and threats for consumer-oriented (wearable) technologies, but does not explicitly distinct between different technologies employed by these devices. Arguably different technologies have different strengths, weaknesses, opportunities and threats, and practitioners should carefully check suitability of their device for their purpose. Finally, literature reviews are inherently dependent on the available literature in the respective context.

## 5 Conclusions

Consumer-oriented (wearable) sleep tracking technologies provide a feasible and scalable approach for longitudinal sleep monitoring in real-world settings and can enhance awareness of sleep-related behaviors while supporting integrated health and performance management. However, their practical utility is constrained by limited validation, lack of transparency in algorithms, and methodological limitations in accurately capturing key sleep parameters, particularly sleep stages. Consequently, data derived from these devices require cautious interpretation and should be contextualized with additional information such as e.g., subjective assessments of sleep. When applied critically and longitudinally, consumer-oriented wearable technology can provide meaningful insights and support self-management of sleep, while uncritical use may lead to misinterpretation, maladaptive behaviors, and inappropriate training or recovery decisions. Future research should prioritize high-quality validation studies, standardized evaluation frameworks, and context-specific applications across populations. Concurrently, user education and transparent reporting standards are essential to ensure responsible implementation and to maximize the benefit–risk ratio of consumer sleep technologies in physically active populations.

## Data Availability

The original contributions presented in the study are included in the article/Supplementary Material, further inquiries can be directed to the corresponding author.

## References

[1] Chandrasekaran B Fernandes S Davis F. Science of sleep and sports performance—a scoping review. Sci Sports. (2020) 35(1):3–11. 10.1016/j.scispo.2019.03.006

[2] Goel N Basner M Rao H Dinges DF. Circadian rhythms, sleep deprivation, and human performance. Prog Mol Biol Transl Sci. (2013) 119:155–90. 10.1016/B978-0-12-396971-2.00007-5 23899598 PMC3963479

[3] Grandner MA. Sleep, health, and society. Sleep Med Clin. (2017) 12(1):1–22. 10.1016/j.jsmc.2016.10.012 28159089 PMC6203594

[4] Charest J Grandner MA. Sleep and athletic performance: impacts on physical performance, mental performance, injury risk and recovery, and mental health. Sleep Med Clin. (2020) 15(1):41–57. 10.1016/j.jsmc.2019.11.005 32005349 PMC9960533

[5] Clemente FM Afonso J Costa J Oliveira R Pino-Ortega J Rico-González M. Relationships between sleep, athletic and match performance, training load, and injuries: a systematic review of soccer players. Healthcare. (2021) 9(7):808. 10.3390/healthcare9070808 34206948 PMC8305909

[6] Fullagar HHK Skorski S Duffield R Hammes D Coutts AJ Meyer T. Sleep and athletic performance: the effects of sleep loss on exercise performance, and physiological and cognitive responses to exercise. Sports Med Auckl NZ. (2015) 45(2):161–86. 10.1007/s40279-014-0260-0 25315456

[7] Craven J McCartney D Desbrow B Sabapathy S Bellinger P Roberts L. Effects of acute sleep loss on physical performance: a systematic and meta-analytical review. Sports Med Auckl NZ. (2022) 52(11):2669–90. 10.1007/s40279-022-01706-y PMC9584849 35708888

[8] Hirshkowitz M Whiton K Albert SM Alessi C Bruni O DonCarlos L. National sleep foundation’s updated sleep duration recommendations: final report. Sleep Health. (2015) 1(4):233–43. 10.1016/j.sleh.2015.10.004 29073398

[9] Biggins M Purtill H Fowler P Bender A Sullivan KO Samuels C. Sleep, health, and well-being in elite athletes from different sports, before, during, and after international competition. Phys Sportsmed. (2021) 49(4):429–37. 10.1080/00913847.2020.1850149 33251907

[10] Gupta L Morgan K Gilchrist S. Does elite sport degrade sleep quality? A systematic review. Sports Med Auckl NZ. (2017) 47(7):1317–33. 10.1007/s40279-016-0650-6 PMC5488138 27900583

[11] Vlahoyiannis A Aphamis G Bogdanis GC Sakkas GK Andreou E Giannaki CD. Deconstructing athletes’ sleep: a systematic review of the influence of age, sex, athletic expertise, sport type, and season on sleep characteristics. J Sport Health Sci. (2021) 10(4):387–402. 10.1016/j.jshs.2020.03.006 32325024 PMC8343120

[12] Bonnar D Bartel K Kakoschke N Lang C. Sleep interventions designed to improve athletic performance and recovery: a systematic review of current approaches. Sports Med Auckl NZ. (2018) 48(3):683–703. 10.1007/s40279-017-0832-x 29352373

[13] Kellmann M Bertollo M Bosquet L Brink M Coutts AJ Duffield R. Recovery and performance in sport: consensus statement. Int J Sports Physiol Perform. (2018) 13(2):240–5. 10.1123/ijspp.2017-0759 29345524

[14] Simpson NS Gibbs EL Matheson GO. Optimizing sleep to maximize performance: implications and recommendations for elite athletes. Scand J Med Sci Sports. (2017) 27(3):266–74. 10.1111/sms.12703 27367265

[15] Walsh NP Halson SL Sargent C Roach GD Nédélec M Gupta L. Sleep and the athlete: narrative review and 2021 expert consensus recommendations. Br J Sports Med. (2020) 55(7):356–68. 10.1136/bjsports-2020-102025 33144349

[16] Rundo JV Downey R III. Polysomnography. in: Handbook of Clinical Neurology. Amsterdam, Oxford, Cambridge: Elsevier (2019). p. 381–92. 10.1016/B978-0-444-64032-1.00025-4 31277862

[17] Kolla BP Mansukhani S Mansukhani MP. Consumer sleep tracking devices: a review of mechanisms, validity and utility. Expert Rev Med Devices. (2016) 13(5):497–506. 10.1586/17434440.2016.1171708 27043070

[18] Trabelsi K BaHammam AS Chtourou H Jahrami H Vitiello MV. The good, the bad, and the ugly of consumer sleep technologies use among athletes: a call for action. J Sport Health Sci. (2023) 12(4):486–8. 10.1016/j.jshs.2023.02.005 36868375 PMC10362482

[19] Fietze I. Sleep applications to assess sleep quality. Sleep Med Clin. (2016) 11(4):461–8. 10.1016/j.jsmc.2016.08.008 28118870

[20] Mansukhani MP Kolla BP. Apps and fitness trackers that measure sleep: are they useful? Cleve Clin J Med. (2017) 84(6):451–6. 10.3949/ccjm.84a.15173 28628429

[21] Chiang AA Khosla S. Consumer wearable sleep trackers: are they ready for clinical use? Sleep Med Clin. (2023) 18(3):311–30. 10.1016/j.jsmc.2023.05.005 37532372

[22] Kim K Park DY Song YJ Han S Kim HJ. Consumer-grade sleep trackers are still not up to par compared to polysomnography. Sleep Breath. (2022) 26(4):1573–82. 10.1007/s11325-021-02493-y 34741243

[23] Roomkham S Lovell D Cheung J Perrin D. Promises and challenges in the use of consumer-grade devices for sleep monitoring. IEEE Rev Biomed Eng. (2018) 11:53–67. 10.1109/RBME.2018.2811735 29993607

[24] Kainec KA Caccavaro J Barnes M Hoff C Berlin A Spencer RMC. Evaluating accuracy in five commercial sleep-tracking devices compared to research-grade actigraphy and polysomnography. Sensors. (2024) 24(2):635. 10.3390/s24020635 38276327 PMC10820351

[25] Düking P Holmberg HC Sperlich B. The potential usefulness of virtual reality systems for athletes: a short SWOT analysis. Front Physiol. (2018) 9:128. 10.3389/fphys.2018.00128 29551978 PMC5841195

[26] Sperlich B Düking P Holmberg HC. A SWOT analysis of the use and potential misuse of implantable monitoring devices by athletes. Front Physiol. (2017) 8:629. 10.3389/fphys.2017.00629 28928670 PMC5591786

[27] Sperlich B Düking P Leppich R Holmberg HC. Strengths, weaknesses, opportunities, and threats associated with the application of artificial intelligence in connection with sport research, coaching, and optimization of athletic performance: a brief SWOT analysis. Front Sports Act Living. (2023) 5:1258562. 10.3389/fspor.2023.1258562 37920303 PMC10618674

[28] Page MJ McKenzie JE Bossuyt PM Boutron I Hoffmann TC Mulrow CD. The PRISMA 2020 statement: an updated guideline for reporting systematic reviews. Br Med J. (2021) 372:n71. 10.1136/bmj.n71 33782057 PMC8005924

[29] Yoon H Choi SH. Technologies for sleep monitoring at home: wearables and nearables. Biomed Eng Lett. (2023) 13(3):313–27. 10.1007/s13534-023-00305-8 37519880 PMC10382403

[30] Halman A Oshlack A. Catchii: empowering literature review screening in healthcare. Res Synth Methods. (2024) 15(1):157–65. 10.1002/jrsm.1675 37771210

[31] Page MJ Sterne JAC Boutron I Hróbjartsson A Kirkham JJ Li T. ROB-ME: a tool for assessing risk of bias due to missing evidence in systematic reviews with meta-analysis. Br Med J. (2023) 383:e076754. 10.1136/bmj-2023-076754 37984978

[32] Sterne JAC Savović J Page MJ Elbers RG Blencowe NS Boutron I. Rob 2: a revised tool for assessing risk of bias in randomised trials. Br Med J. (2019) 366:l4898. 10.1136/bmj.l4898 31462531

[33] Sterne JA Hernán MA Reeves BC Savović J Berkman ND Viswanathan M. ROBINS-I: a tool for assessing risk of bias in non-randomised studies of interventions. Br Med J. (2016) 355:i4919. 10.1136/bmj.i4919 27733354 PMC5062054

[34] Charmaz K. Constructing Grounded Theory: A Practical Guide through Qualitative Analysis (2006).

[35] Corbin J Strauss A. Basics of Qualitative Research (3rd ed.): Techniques and Procedures for Developing Grounded Theory. London, Thousand Oaks, New Delhi: SAGE Publications, Inc (2008).

[36] Saldana J. The Coding Manual for Qualitative Researchers. 5th ed London, Thousand Oaks, New Delhi: SAGE Publications (2025).

[37] Claudino JG Gabbet TJ de Sá Souza H Simim M Fowler P de Alcantara Borba D. Which parameters to use for sleep quality monitoring in team sport athletes? A systematic review and meta-analysis. BMJ Open Sport Exerc Med. (2019) 5(1):000475. 10.1136/bmjsem-2018-000475 PMC6340585 30729029

[38] Cameron M Bird SP. Sleep monitoring in elite athletes: a brief review of smartphone applications and recommendations. J Aust Strength Cond .(2015) 23(5):62–72.

[39] Cole T. Sleep tracking: a review of the use of technology to monitor sleep in elite populations. J Aust Strength Cond. (2017) 25(2):75–81.

[40] de Zambotti M Cellini N Menghini L Sarlo M Baker FC. Sensors capabilities, performance, and use of consumer sleep technology. Sleep Med Clin. (2020) 15(1):1–30. 10.1016/j.jsmc.2019.11.003 32005346 PMC7482551

[41] Halson SL. Sleep monitoring in athletes: motivation, methods, miscalculations and why it matters. Sports Med Auckl NZ. (2019) 49(10):1487–97. 10.1007/s40279-019-01119-4 31093921

[42] Driller MW Dunican IC Omond SET Boukhris O Stevenson S Lambing K. Pyjamas, polysomnography and professional athletes: the role of sleep tracking technology in sport. Sports. (2023) 11(1):14. 10.3390/sports11010014 36668718 PMC9861232

[43] Lujan MR Perez-Pozuelo I Grandner MA. Past, present, and future of multisensory wearable technology to monitor sleep and circadian rhythms. Front Digit Health. (2021) 3:721919. 10.3389/fdgth.2021.721919 34713186 PMC8521807

[44] Mathunjwa BM Kor RYJ Ngarnkuekool W Hsu YL. A comprehensive review of home sleep monitoring technologies: smartphone apps, smartwatches, and smart mattresses. Sensors. (2025) 25(6):1771. 10.3390/s25061771 40292882 PMC11945902

[45] Nobari H Banihashemi M Saedmocheshi S Prieto-González P Oliveira R. Overview of the impact of sleep monitoring on optimal performance, immune system function and injury risk reduction in athletes: a narrative review. Sci Prog. (2023) 106(4):00368504231206265. 10.1177/00368504231206265 37990537 PMC10666701

[46] Peake JM Kerr G Sullivan JP. A critical review of consumer wearables, Mobile applications, and equipment for providing biofeedback, monitoring stress, and sleep in physically active populations. Front Physiol. (2018) 9:743. 10.3389/fphys.2018.00743 30002629 PMC6031746

[47] Seshadri DR Thom ML Harlow ER Gabbett TJ Geletka BJ Hsu JJ. Wearable technology and analytics as a complementary toolkit to optimize workload and to reduce injury burden. Front Sports Act Living. (2021) 2:630576. 10.3389/fspor.2020.630576 33554111 PMC7859639

[48] Vlahoyiannis A Sakkas GK Manconi M Aphamis G Giannaki CD. A critical review on sleep assessment methodologies in athletic populations: factors to be considered. Sleep Med. (2020) 74:211–23. 10.1016/j.sleep.2020.07.029 32861013

[49] Jakowski S Stork M. Effects of sleep self-monitoring via app on subjective sleep markers in student athletes. Somnologie (Berl). (2022) 26(4):244–51. 10.1007/s11818-022-00395-z 36311283 PMC9595090

[50] Fuller KL Juliff L Gore CJ Peiffer JJ Halson SL. Software thresholds alter the bias of actigraphy for monitoring sleep in team-sport athletes. J Sci Med Sport. (20171) 20(8):756–60. 10.1016/j.jsams.2016.11.021 28189461

[51] Jakowski S. Self-tracking via smartphone app: potential tool for athletes’ recovery self-management? Ger J Exerc Sport Res. (2022) 52(2):253–61. 10.1007/s12662-022-00812-3 40477594 PMC9053116

[52] Gooderick J Wood T Abbott W Hayes M Maxwell N. Does a self-reported sleep duration reflect actigraphy reported sleep duration in female football players? Sci Med Footb. (2025) 9(1):19–25. 10.1080/24733938.2023.2297903 38174382

[53] Hof Zum Berge A Kellmann M Kallweit U Mir S Gieselmann A Meyer T. Portable PSG for sleep stage monitoring in sports: assessment of SOMNOwatch plus EEG. Eur J Sport Sci. (2020) 20(6):713–21. 10.1080/17461391.2019.1659421 31456506

[54] Kawasaki Y Kasai T Sakurama Y Sekiguchi A Kitamura E Midorikawa I. Evaluation of sleep parameters and sleep staging (slow wave sleep) in athletes by fitbit Alta HR, a consumer sleep tracking device. Nat Sci Sleep. (2022) 14:819–27. 10.2147/NSS.S351274 35502231 PMC9056106

[55] Nuuttila OP Schäfer Olstad D Martinmäki K Uusitalo A Kyröläinen H. Monitoring sleep and nightly recovery with wrist-worn wearables: links to training load and performance adaptations. Sensors. (2025) 25(2):533. 10.3390/s25020533 39860902 PMC11768492

[56] Peacock CA Mena M Sanders GJ Silver TA Kalman D Antonio J. Sleep data, physical performance, and injuries in preparation for professional mixed martial arts. Sports. (2019) 7(1):1. 10.3390/sports7010001 PMC6359324 30577414

[57] Roach GD Miller DJ Shell SJ Miles KH Sargent C. Validation of a neurophysiological-based wearable device (somfit) for the assessment of sleep in athletes. Sensors. (2025) 25(7):2123. 10.3390/s25072123 40218633 PMC11991079

[58] Wick AZ Combertaldi SL Rasch B. The first-night effect of sleep occurs over nonconsecutive nights in unfamiliar and familiar environments. Sleep. (2024) 47(10):zsae179. 10.1093/sleep/zsae179 39126649 PMC11467056

[59] De Fazio R Mastronardi VM De Vittorio M Visconti P. Wearable sensors and smart devices to monitor rehabilitation parameters and sports performance: an overview. Sensors. (2023) 23(4):1856. 10.3390/s23041856 36850453 PMC9965388

[60] Düking P Hotho A Holmberg HC Fuss FK Sperlich B. Comparison of non-invasive individual monitoring of the training and health of athletes with commercially available wearable technologies. Front Physiol. (2016) 7:71. 10.3389/fphys.2016.00071 27014077 PMC4783417

[61] Klier K Seiler K Wagner M. On the usability of digital sleep interventions in sports. Ger J Exerc Sport Res. (2022) 52(3):482–5. 10.1007/s12662-021-00771-1

[62] Ancoli-Israel S Martin JL Blackwell T Buenaver L Liu L Meltzer LJ. The SBSM guide to actigraphy monitoring: clinical and research applications. Behav Sleep Med. (2015) 13(Suppl 1):4–38. 10.1080/15402002.2015.1046356 26273913

[63] Lee T Cho Y Cha KS Jung J Cho J Kim H. Accuracy of 11 wearable, nearable, and airable consumer sleep trackers: prospective multicenter validation study. JMIR MHealth UHealth. (2023) 11(1):e50983. 10.2196/50983 37917155 PMC10654909

[64] Doherty C Baldwin M Keogh A Caulfield B Argent R. Keeping pace with wearables: a living Umbrella review of systematic reviews evaluating the accuracy of consumer wearable technologies in health measurement. Sports Med. (2024) 54(11):2907–26. 10.1007/s40279-024-02077-2 39080098 PMC11560992

[65] Ibáñez V Silva J Navarro E Cauli O. Sleep assessment devices: types, market analysis, and a critical view on accuracy and validation. Expert Rev Med Devices. (2019) 16(12):1041–52. 10.1080/17434440.2019.1693890 31774330

[66] de Zambotti M Goldstein C Cook J Menghini L Altini M Cheng P. State of the science and recommendations for using wearable technology in sleep and circadian research. Sleep. (2024) 47(4):zsad325. 10.1093/sleep/zsad325 38149978

[67] Düking P Fuss FK Holmberg HC Sperlich B. Recommendations for assessment of the reliability, sensitivity, and validity of data provided by wearable sensors designed for monitoring physical activity. JMIR MHealth UHealth. (2018) 6(4):e9341. 10.2196/mhealth.9341 PMC5952119 29712629

[68] Robertson S Zendler J De Mey K Haycraft J Ash GI Brockett C. Development of a sports technology quality framework. J Sports Sci. (2023) 41(22):1983–93. 10.1080/02640414.2024.2308435 38305379

[69] Perez-Pozuelo I Zhai B Palotti J Mall R Aupetit M Garcia-Gomez JM. The future of sleep health: a data-driven revolution in sleep science and medicine. NPJ Digit Med. (2020) 3:42. 10.1038/s41746-020-0244-4 32219183 PMC7089984

[70] Devrim-Lanpir A Devenney S Egan B. Evaluation of the agreement between research-grade actigraphy sleep, consumer-grade smartwatches and self-reported sleep diaries in masters endurance athletes. J Sleep Res. (2025) 34(6):e70177. 10.1111/jsr.70177 40851489 PMC12592815

[71] Birrer V Elgendi M Lambercy O Menon C. Evaluating reliability in wearable devices for sleep staging. Npj Digit Med. (2024) 7(1):74. 10.1038/s41746-024-01016-9 38499793 PMC10948771

[72] Chinoy ED Cuellar JA Huwa KE Jameson JT Watson CH Bessman SC. Performance of seven consumer sleep-tracking devices compared with polysomnography. Sleep. (2021) 44(5):zsaa291. 10.1093/sleep/zsaa291 33378539 PMC8120339

[73] Baranwal N Yu PK Siegel NS. Sleep physiology, pathophysiology, and sleep hygiene. Prog Cardiovasc Dis. (2023) 77:59–69. 10.1016/j.pcad.2023.02.005 36841492

[74] de Gans CJ Burger P van den Ende ES Hermanides J Nanayakkara PWB Gemke RJBJ. Sleep assessment using EEG-based wearables—a systematic review. Sleep Med Rev. (2024) 76:101951. 10.1016/j.smrv.2024.101951 38754209

[75] Hung SH Serwa K Rosenthal G Eng JJ. Validity of heart rate measurements in wrist-based monitors across skin tones during exercise. PLoS One. (2025) 20(2):e0318724. 10.1371/journal.pone.0318724 39928630 PMC11809914

[76] Koerber D Khan S Shamsheri T Kirubarajan A Mehta S. Accuracy of heart rate measurement with wrist-worn wearable devices in Various skin tones: a systematic review. J Racial Ethn Health Disparities. (2023) 10(6):2676–84. 10.1007/s40615-022-01446-9 36376641 PMC9662769

[77] Doherty C Baldwin M Lambe R Altini M Caulfield B. Privacy in consumer wearable technologies: a living systematic analysis of data policies across leading manufacturers. Npj Digit Med. (2025) 8(1):363. 10.1038/s41746-025-01757-1 40517175 PMC12167361

[78] Genaro Motti V Feng H. Wearable privacy. In: Mahato K, Pandya A, editors. Progress in Molecular Biology and Translational Science. Cambridge, London: Academic Press (2025). p. 27–48. 10.1016/bs.pmbts.2025.06.005 40921537

[79] Robbins R Grandner MA Buxton OM Hale L Buysse DJ Knutson KL. Sleep myths: an expert-led study to identify false beliefs about sleep that impinge upon population sleep health practices. Sleep Health. (2019) 5(4):409–17. 10.1016/j.sleh.2019.02.002 31003950 PMC6689426

[80] Bragazzi NL Garbarino S. The Complex interaction between sleep-related information, misinformation, and sleep health: call for comprehensive research on sleep infodemiology and infoveillance. JMIR Infodemiology. (2024) 4(1):e57748. 10.2196/57748 39475424 PMC11681283

[81] Borle P Reichel K Niebuhr F Voelter-Mahlknecht S. How are techno-stressors associated with mental health and work outcomes? A systematic review of occupational exposure to information and communication technologies within the technostress model. Int J Environ Res Public Health. (2021) 18(16):8673. 10.3390/ijerph18168673 34444422 PMC8394886

[82] Werner M Bischof A. The double-edged sword of self-tracking: investigating factors of technostress in performance-oriented cycling and triathlon. Front Sports Act Living. (2024) 6:1465515. 10.3389/fspor.2024.1465515 39624622 PMC11608990

[83] Baron KG Abbott S Jao N Manalo N Mullen R. Orthosomnia: are some patients taking the quantified self too far? J Clin Sleep Med. (2017) 13(2):351–4. 10.5664/jcsm.6472 27855740 PMC5263088

[84] Jahrami H Trabelsi K Husain W Ammar A BaHammam AS Pandi-Perumal SR. Prevalence of orthosomnia in a general population sample: a cross-sectional study. Brain Sci. (2024) 14(11):1123. 10.3390/brainsci14111123 39595886 PMC11592250

[85] Chee MWL Baumert M Scott H Cellini N Goldstein C Baron K. World sleep society recommendations for the use of wearable consumer health trackers that monitor sleep. Sleep Med. (2025) 131:106506. 10.1016/j.sleep.2025.106506 40300398

[86] Shei RJ Holder IG Oumsang AS Paris BA Paris HL. Wearable activity trackers–advanced technology or advanced marketing? Eur J Appl Physiol. (2022) 122(9):1975–90. 10.1007/s00421-022-04951-1 35445837 PMC9022022

[87] Windt J MacDonald K Taylor D Zumbo BD Sporer BC Martin DT. To tech or not to tech?” A critical decision-making framework for implementing technology in sport. J Athl Train. (2020) 55(9):902–10. 10.4085/1062-6050-0540.19 32991702 PMC7534935

[88] Malode SJ Alshehri MA Shetti NP. Revolutionizing human healthcare with wearable sensors for monitoring human strain. Colloids Surf B Biointerfaces. (2025) 246:114384. 10.1016/j.colsurfb.2024.114384 39579495

[89] Ye S Feng S Huang L Bian S. Recent progress in wearable biosensors: from healthcare monitoring to sports analytics. Biosensors. (2020) 10(12):205. 10.3390/bios10120205 33333888 PMC7765261

[90] Hu X Sgherza TR Nothrup JB Fresco DM Naragon-Gainey K Bylsma LM. From lab to life: evaluating the reliabilityand validity of psychophysiological data from wearable devices in laboratory andambulatory settings. Behav Res Methods. (2024) 56(7):1–20. 10.3758/s13428-024-02387-3 38528248 PMC12403079

[91] Moorthy P Weinert L Schüttler C Svensson L Sedlmayr B Müller J. Attributes, methods, and frameworks used to evaluate wearables and their companion mHealth apps: scoping review. JMIR MHealth UHealth. (2024) 12(1):e52179. 10.2196/52179 38578671 PMC11031706

[92] Schoenmakers M Saygin M Sikora M Vaessen T Noordzij M de Geus E. Stress in action wearables database: a database of noninvasive wearable monitors with systematic technical, reliability, validity, and usability information. Behav Res Methods. (2025) 57(6):171. 10.3758/s13428-025-02685-4 40360861 PMC12075381

[93] Schembre SM Liao Y Robertson MC Dunton GF Kerr J Haffey ME. Just-in-Time feedback in diet and physical activity interventions: systematic review and practical design framework. J Med Internet Res. (2018) 20(3):e8701. 10.2196/jmir.8701 PMC5887039 29567638

[94] Van Hooren B Goudsmit J Restrepo J Vos S. Real-time feedback by wearables in running: current approaches, challenges and suggestions for improvements. J Sports Sci. (2020) 38(2):214–30. 10.1080/02640414.2019.1690960 31795815

[95] Baumann MF Weinberger N Maia M Schmid K. User types, psycho-social effects and societal trends related to the use of consumer health technologies. Digit Health. (2023) 9:20552076231163996. 10.1177/20552076231163996 37034307 PMC10074638

[96] Merrill MA Paruchuri A Rezaei N Kovacs G Perez J Liu Y. Transforming wearable data into personal health insights using large language model agents. Nat Commun. (2026) 17(1):1143. 10.1038/s41467-025-67922-y 41526380 PMC12855967

[97] Suzuki C Suzuki Y Abe T Kanbayashi T Fukusumi S Kokubo T. Mobile sleep lab: comparison of polysomnographic parameters with a conventional sleep laboratory. PLoS One. (2025) 20(1):e0316579. 10.1371/journal.pone.0316579 39775303 PMC11706495

[98] Pantesco EJ Kan IP. False beliefs about sleep and their associations with sleep-related behavior. Sleep Health. (2022) 8(2):216–24. 10.1016/j.sleh.2021.10.004 34840105

[99] Düking P Robertson S Holmberg HC Wolf KH Sperlich B. Classification system for ai-enabled consumer-grade wearable technologies aiming to automatize decision-making about individualization of exercise procedures. Front Sports Act Living. (2025) 6:1500563. 10.3389/fspor.2024.1500563 39945004 PMC11813931

[100] Halson SL Peake JM Sullivan JP. Wearable technology for athletes: information overload and pseudoscience? Int J Sports Physiol Perform. (2016) 11(6):705–6. 10.1123/IJSPP.2016-0486 27701967

[101] de Zambotti M Cellini N Goldstone A Colrain IM Baker FC. Wearable sleep technology in clinical and research settings. Med Sci Sports Exerc. (2019) 51(7):1538. 10.1249/MSS.0000000000001947 30789439 PMC6579636

[102] Mogavero MP Lanza G Bruni O Ferini-Strambi L Silvani A Faraguna U. Beyond the sleep lab: a narrative review of wearable sleep monitoring. Bioengineering. (2025) 12(11):1191. 10.3390/bioengineering12111191 41301147 PMC12649296

[103] Trimmel K Eder HG Böck M Stefanic-Kejik A Klösch G Seidel S. The (mis)perception of sleep: factors influencing the discrepancy between self-reported and objective sleep parameters. J Clin Sleep Med. (2021) 17(5):917–24. 10.5664/jcsm.9086 33393901 PMC8320481

[104] Cho S Ensari I Weng C Kahn MG Natarajan K. Factors affecting the quality of person-generated wearable device data and associated challenges: rapid systematic review. JMIR MHealth UHealth. (2021) 9(3):e20738. 10.2196/20738 33739294 PMC8294465

[105] Woolley SI Collins T Mitchell J Fredericks D. Investigation of wearable health tracker version updates. BMJ Health Care Inform. (2019) 26(1):e100083. 10.1136/bmjhci-2019-100083 31597642 PMC7062347

[106] Giurgiu M von Haaren-Mack B Fiedler J Woll S Burchartz A Kolb S. The wearable landscape: issues pertaining to the validation of the measurement of 24-h physical activity, sedentary, and sleep behavior assessment. J Sport Health Sci. (2025) 14:101006. 10.1016/j.jshs.2024.101006 39491744 PMC11809201

