# Supplementary material for: Consumer-oriented (wearable) sleep technology: a systematic SWOT analysis and recommendations for athletes and fitness enthusiasts
Source: Front Sports Act Living. 2026 Jul 10;8:1872518. doi: 10.3389/fspor.2026.1872518 (PMC13395882; doi:10.3389/fspor.2026.1872518)
Supplement: Supplementary file 1 [file Table1.docx]

Supplementary Material

**Supplementary Table S1.** Overview of the included studies.

|  | Author(s) | Year | Study Type | Population | Context / Device | Parameters / Measures | Outcome | Risk of Bias |
| --- | --- | --- | --- | --- | --- | --- | --- | --- |
| 1 | Cameron & Bird | 2015 | Review + Recommendations | elite athletes | smartphone applications | total time in bed, total time asleep, sleep efficiency, sleep onset latency, number of awakenings, deep/light sleep stages | Sleep apps for monitoring + providing feedback to optimize sleep hygiene and enhance recovery. Coach perspective: practical + effective approach for early detection of overtraining symptoms and underrecovery in athletes | NA |
|  |  |  |  |  |  |  | Only few sleep apps are validated/compared to actigraphy (e.g., Sleep, Sleep Cycle, Sleep MotionX, Sleepful App). |  |
|  |  |  |  |  |  |  | Commercialized sleep monitoring applications designed either for Android or iOS. |  |
| 2 | Cole | 2017 | Review | elite athletic populations | monitoring technology | time in bed, time asleep, sleep efficiency, moving time | Actigraphy as free-living gold standard which is enhanced when combined with subjective reports (e.g., sleep logs). | NA |
|  |  |  |  |  |  |  | Different equations/algorithms used by different devices 🡪 limited accuracy |  |
| 3 | Fuller et al. | 2017 | Original Research (part of RCT; free-living validation, gold standard criterion measure) | team-sport athletes (N = 21 male Australian Football + Rugby players, 22.5 ± 2.7 years old) | actigraphy | total sleep time, sleep efficiency, wake after sleep onset, wake bouts, sleep onset latency | Actical® actigraphy as reliable, wrist-worn, low-cost, non-invasive alternative for collecting sleep data in free-lining environment. Actiware® software allows data processing with different sleep-wake thresholds. | low |
|  |  |  |  |  |  |  | Over-/underestimation of sleep parameters depending on applied threshold. For team sport: Medium threshold (activity counts above 40) recommended |  |
| 4 | Peake et al. | 2018 | Review | physically active populations | consumer wearables + mobile applications | hydration status, metabolism, training loads, movement patterns, injury risks, heart rate, heart rate variability, breathing patterns, sleep, stress, cognitive functions | Consumer considerations when choosing a technology: type of device, provided outcomes, availability, validity, usability | NA |
|  |  |  |  |  |  |  | Most of consumer technologies are not labeled as medical devices, but support the individuals' overall health awareness. |  |
| 5 | Claudino et al. | 2019 | Systematic Review + Meta-Analysis | team sport athletes | monitoring parameters + tools | sleep efficiency, sleep latency, time awake, time asleep, bed time, moving time | Definition & calculation of sleep parameters can differ between monitoring tools. | low |
|  |  |  |  |  |  |  | Actigraphy for objective assessment + diaries/scales commonly used for subjective assessment. Sustainable sleep monitoring should be user-friendly, low cost and measure a wide range of parameters in several contexts. |  |
| 6 | Halson | 2019 | Review | athletes | monitoring tools + methods | sleep efficiency, sleep duration, wake after sleep onset, sleep latency, light/deep sleep stages | Available sleep monitoring tools: polysomnography, actigraphy, wearable + nearable consumer technology, smartphone applications, diaries + questionnaires | NA |
|  |  |  |  |  |  |  | Regular monitoring recommended (important for training and health state). Daily monitoring or regular 1-/2-week monitoring period and interpretation of sleep data in consideration of the athlete's typical 24h |  |
| 7 | Peacock et al. | 2019 | Original Research (free-living) | professional MMA athletes (N = 8 male Mixed Martial Artists, 27.7 ± 3.4 years old) | wrist-worn wearable | sleep latency, sleep efficiency, onset, wake variances | Readiband enabled to maintain individual sleep routine/behavior. | serious |
|  |  |  |  |  |  |  | Consistent sleep schedule beneficial for performance improvements and injury reduction |  |
| 8 | de Zambotti et al. | 2020 | Review | general population | sensor capabilities + consumer technology | sleep/wake patterns, heart rate, heart rate variability, respiration pattern, motion | Growing number of sensors in consumer sleep technologies. Higher accuracy in multisensory devices. Most common features for sleep tracking based on HR/HRV, respiratory pattern, motion. Lowest evidence/validity of consumer wearables in tracking sleep stages. | NA |
|  |  |  |  |  |  |  | Summary of sleep data available via dedicated mobile app or Internet platform (often no access to raw data for users). Third-party services limit data security. |  |
| 9 | Hof zum Berge et al. | 2020 | Original Research (lab validation, gold standard criterion measure) | athletes / physically active population (N = 25, 40 % male, 22.92 ± 2.03 years old) | portable PSG | sleep onset latency, wake after sleep onset, total wake time, total sleep time, sleep efficiency, sleep stages | Somnowatch plus EEG as economical and time-friendly alternative to activity-based devices. EEG electrodes allow assessment of sleep architecture and stages. | low |
|  |  |  |  |  |  |  | Although the portable PSG provides more information than actigraphy, it does not replace in-lab diagnostics in case of clinically relevant sleep problems. |  |
| 10 | Vlahoyiannis et al. | 2020 | Review | athletes | assessment methodologies | total sleep time, sleep onset latency, sleep efficiency, wake periods, sleep stages | Factors to consider when applying sleep tracking technology: accuracy, sleep data access, invasive, compliance, expertise required, clinical use, use in athletes, cost | NA |
|  |  |  |  |  |  |  | For holistic insights tracking of sleep-related, nutrition, and lifestyle factors recommended. Assessment methods in athletes: polysomnography, actigraphy, wearable/nearable consumer technologies, apps, self-reports |  |
| 11 | Lujan et al. | 2021 | Review | general population | multisensory wearable technology | sleep/wake timing, total sleep time, sleep latency, wake after sleep onset, sleep efficiency | Actiwatch, Mini-Mitter, Motionlogger, ActiGraph GT3Xx as the most validated actigraphs; Fitbit trackers, Apple Watches, Oura rings as multisensory/next-generation consumer wearables | NA |
|  |  |  |  |  |  |  | Other categories of consumer sleep technology: phone-based accelerometers, cardioballistic sensors, beside sensors, in-bed sensors, wearable/portable EEG devices. Limitations due to lack of validity and recording metrics. |  |
| 12 | Seshadri et al. | 2021 | Narrative Review | athletes | wearable technology | heart rate, heart rate variability, muscle oxygen saturation, respiration rate, acceleration, hydration, sleep, load, movement profiles | Often used wearable technologies in sports: EMG, GPS, accelerometers, ECG/PPG sensors (e.g., Catapult, Zebra, Moxy, Whoop, Fitbit, Apple). Wearable data as support for decision-making and expertise of athletes + coaches. | NA |
|  |  |  |  |  |  |  | Actigraphy devices are the most widely used sleep sensors 🡪 due to their reliance on motion to assess sleep they tend to overestimate sleep und underestimate wakefulness across sleep periods (i.e., high sensitivity but low specificity for sensing sleep). Machine Learning approaches seem beneficial for higher accuracy of devices. |  |
| 13 | Jakowski | 2022 | Original Research (online survey) | athletes (N = 217, 71 % male, 26.9 ± 7 years old) | smartphone app | fitness, nutrition, sleep | Different stages of engagement in users of tracking apps/devices. Individuals who already track their training or nutrition seem more likely to also track their sleep. | NA |
|  |  |  |  |  |  |  | Smartphone apps can raise awareness and promote healthy sleep habits. Tracking as support of sleep self-management. |  |
| 14 | Jakowski & Stork | 2022 | Original Research (RCT, free-living) | student athletes (N = 98, 38 % male, 21 ± 1.7 years old) | app | time in bed, total sleep time, sleep efficiency, planned bedtime vs. actual time of lights out | Sleep Score & Sleep Cycle differ in provided feedback and calculated sleep variables (e.g., scoring vs. more graphical summary of a night). | low |
|  |  |  |  |  |  |  | Subjectively rated low agreement between perceived sleep and apps' feedback. |  |
| 15 | Kawasaki et al. | 2022 | Original Research (free-living validation, no gold standard criterion measure) | college athletes (N = 36 Basketball + Track-and-Field athletes, 47 % male, 21.0 ± 0.9 years old) | consumer tracking device | total sleep time, sleep onset latency, wake after sleep onset, sleep stages | ZA-9 as research-grade portable two channel EEG device. Allows reliable sleep staging in home environment, but only feasible over a limited period. Consumer wearables are less labor-intrusive, low cost, and user-friendly. Easy applicable over longer monitoring periods. | moderate |
|  |  |  |  |  |  |  | Fitbit Alta HR detects sleep based on acceleration and heart rate data, but tends to underestimate sleep parameters. Consumer wearables can be part of tailored sleep interventions aiming to optimize the athlete's sleep environment, and thus, improve recovery + performance. |  |
| 16 | Driller et al. | 2023 | Narrative Review | professional athletes | tracking technology | sleep onset latency, sleep onset time, total sleep time, wake after sleep onset, wake time, sleep efficiency, time in bed, sleep onset variance, wake variance | Technological advances lead to "explosion" of available consumer devices for sleep tracking 🡪 usually small devices placed on wrist, finger, head, or chest (e.g., Readiband, Fitbit, ActiGraph, ActiWatch, Whoop, Oura, Garmin, Apple Watch). | NA |
|  |  |  |  |  |  |  | Application + goal of sleep monitoring will inform which sleep tracking method should be used. Integration of PPG sensors in wearables increased accuracy, however, specificity is limited for devices that do not record brain activity. Expertise is needed to interact + interpret the data to make sense how it can be used to improve sleep. |  |
| 17 | Nobari et al. | 2023 | Narrative Review | athletes | monitoring impact | sleep efficiency, sleep time, awakenings, sleep stages, nutrition, load, inflammation | Amount & quality of sleep needed vary depending on age, sex, type + level of sport. Sleep monitoring during training + competition periods. Use of devices or self-reports in dependence of athletes' commitment. | NA |
|  |  |  |  |  |  |  | High complexity of analyzing sleep patterns + high individuality in sleep behavior and health-/performance-related outcomes. With regard to important role of sleep for health + performance, sleep monitoring should get the same attention as load monitoring. |  |
| 18 | Gooderick et al. | 2025 | Original Research (free-living) | female football players (N = 22 female footballers, 19.5 ± 1.3 years old) | self-report + actigraphy | sleep duration | Self-reports as retrospective assessment might be inaccurate due to difficulties of remembering the last night. | moderate |
|  |  |  |  |  |  |  | ActiGraph GT9X as research-grade wearable device. Wearing a device + tracking sleep could be an additional stress for some individuals. Role of tracking + handling of data should be transparent communicated between coaches + athletes. |  |
| 19 | Mathunjwa et al. | 2025 | Review | general population | smartphone apps + smartwatches + smart mattresses | sleep, heart rate, oxygen saturation, respiration rate, movement, menstrual activity, temperature, pressure | Home-based sleep monitoring technologies: smartphone applications, wearables/smartwatches, smart mattresses. Each employs distinct methods for tracking sleep patterns ranging from motion and heart rate sensors to advanced AI algorithms for sleep-stage classification. | NA |
|  |  |  |  |  |  |  | Wearables provide best balance of accuracy, affordability + usability; apps are cost-effective, but less accurate; smart mattresses are costlier + at least validated, but passive + comfortable. |  |
| 20 | Nuuttila et al. | 2025 | Original Research (free-living) | recreational athletes (N = 24 runners, 42 % male, 38.6 ± 6.5 years old) | wrist-worn wearables | sleep time, actual sleep, sleep continuity, sleep score, sleep charge, HR, RMSSD, breathing rate, ANS charge | Polar Vantage V2 provides feedback on algorithm-based metrics (e.g., sleep score, readiness score) as consumers may not have time + skills to interpret raw data. | serious |
|  |  |  |  |  |  |  | Subjective recovery metrics impaired by intensified training, no consistent changes in sleep + nightly recovery metrics. Monitoring nightly recovery to recognize individual responses to training. |  |
| 21 | Roach et al. | 2025 | Original Research (lab validation, gold standard criterion measure) | athletes (N = 27, 48 % male, 22.3 ± 5.1 years old) | neurophysiological-based wearable device | sleep time, wake time, sleep stages | Somfit as neurophysiological-based wearable (EEG forehead patch) seem to have a higher level of agreement with PSG than activity-based wearables. | low |
|  |  |  |  |  |  |  | Headbands + head patches might be less comfortable to wear for daily monitoring compared to finger or wrist-worn devices. |  |

NA = Not Applicable.
